# “ARE YOU BREASTFEEDING?” A SURVEY STUDY ON CAREGIVERS PERCEPTIONS OF HEALTHCARE PROVIDERS LEVEL OF SUPPORT FOR INFANT FEEDING METHODS:, PRESSURE TO BREASTFEED AND CAREGIVERS EMOTIONAL RESPONSE

**DOI:** 10.51894/001c.123125

**Published:** 2024-08-31

**Authors:** Maya Wyrzykowski

**Affiliations:** 1 College of Osteopathic Medicine Michigan State University

## 96

### INTRODUCTION

Healthcare professionals play an integral role in providing education for parents, but self-report a lack of confidence in troubleshooting infant feeding problems. Parents who have negative healthcare experiences, including pressure to breastfeed, and lack of support have been found to have an increased risk of postpartum depression.

### OBJECTIVE

The primary objective of the study was to determine how caregivers perceive a variety of healthcare professionals in terms of support of infant feeding, pressure to breastfeed, and emotional response to interactions.

### METHODS

A survey was distributed over social media, and after exclusion criteria n = 293. The survey collected data on type of healthcare provider, levels of support and pressure, and the associated emotions with each interaction. The same questions were asked in non-healthcare settings to control for societal expectations. Levels of pressure and support were measured on a sliding scale. To measure emotions for interactions respondents chose the top 3 emotions from a list of positive, negative, and neutral emotions. The survey also asked about feeding experience and preferences, socioeconomic, and demographic characteristics.

### RESULTS

Level of perceived support in healthcare settings was greater than societal settings (M = 73.04, SD = 18.86) vs (M = 68.94, SD = 22.70); t(243)=3.08, p=0.001. There was no significant difference in level of pressure to breastfeed between healthcare or societal settings (37.12 vs 37.9, p=0.59). Higher levels of support predicted more positive emotions. Increased level of pressure was associated with less positive emotions. Level of support had a significant impact on the reported emotional response to the conversation (R2 = 0.22, F = 286, p<0.0001). Pressure was found to have a similar impact (R2 = 0.18, F = 219, p<0.0001).

### CONCLUSION

While healthcare providers as a whole provided more support for infant feeding, pressure to breastfeed was ubiquitous both in the clinic setting and society at large. The more a healthcare provider can offer support while subsequently decreasing the pressure to breastfeed, the higher chance of a parent reporting a positive experience, leading to potential decreased risk for postpartum depression.

